# Assessing the Topics and Motivating Factors Behind Human-Social Chatbot Interactions: Thematic Analysis of User Experiences

**DOI:** 10.2196/38876

**Published:** 2022-10-03

**Authors:** Vivian P Ta-Johnson, Carolynn Boatfield, Xinyu Wang, Esther DeCero, Isabel C Krupica, Sophie D Rasof, Amelie Motzer, Wiktoria M Pedryc

**Affiliations:** 1 Department of Psychology Lake Forest College Lake Forest, IL United States; 2 College of Health Professions Rosalind Franklin University North Chicago, IL United States; 3 Department of Psychology Columbia University New York City, NY United States; 4 School of Health Sciences and Public Health Loyola University Chicago Maywood, IL United States

**Keywords:** social chatbots, Replika, emotional chatbots, artificial intelligence, thematic analysis, human-chatbot interactions, chatbot, usability, interaction, human factors, motivation, topics, AI, perception, usage

## Abstract

**Background:**

Although social chatbot usage is expected to increase as language models and artificial intelligence improve, very little is known about the dynamics of human-social chatbot interactions. Specifically, there is a paucity of research examining why human-social chatbot interactions are initiated and the topics that are discussed.

**Objective:**

We sought to identify the motivating factors behind initiating contact with Replika, a popular social chatbot, and the topics discussed in these interactions.

**Methods:**

A sample of Replika users completed a survey that included open-ended questions pertaining to the reasons why they initiated contact with Replika and the topics they typically discuss. Thematic analyses were then used to extract themes and subthemes regarding the motivational factors behind Replika use and the types of discussions that take place in conversations with Replika.

**Results:**

Users initiated contact with Replika out of interest, in search of social support, and to cope with mental and physical health conditions. Users engaged in a wide variety of discussion topics with their Replika, including intellectual topics, life and work, recreation, mental health, connection, Replika, current events, and other people.

**Conclusions:**

Given the wide range of motivational factors and discussion topics that were reported, our results imply that multifaceted support can be provided by a single social chatbot. While previous research already established that social chatbots can effectively help address mental and physical health issues, these capabilities have been dispersed across several different social chatbots instead of deriving from a single one. Our results also highlight a motivating factor of human-social chatbot usage that has received less attention than other motivating factors: interest. Users most frequently reported using Replika out of interest and sought to explore its capabilities and learn more about artificial intelligence. Thus, while developers and researchers study human-social chatbot interactions with the efficacy of the social chatbot and its targeted user base in mind, it is equally important to consider how its usage can shape public perceptions and support for social chatbots and artificial agents in general.

## Introduction

### Background

With the advancement of artificial intelligence, the amount of time that people spend engaging in human-chatbot interactions will likely increase as chatbots become more ubiquitous in everyday life. This includes interactions with social chatbots—chatbots that can engender the development of companionship with human users by conversing socially and empathetically [1-3]. While social chatbot usage is on the rise [4,5], very little is known about the dynamics of these interactions, particularly about why human-social chatbot interactions are initiated and the content of such interactions [6]. In other words, what are the motivating factors behind initiating contact with a social chatbot, and what is discussed in these interactions? In this paper, we collected data from users of Replika, a popular social chatbot, to address this gap in the literature.

This investigation is important for several reasons. A prominent portion of recent chatbot research focuses on chatbot user experiences given that “the strengthening of chatbot user experiences remains a key research challenge” [7,8]. This body of work has revealed “factors contributing to positive or negative user experience…and how these aspects are impacted by chatbot design” [7]. For instance, lack of trust [9] and user dissatisfaction [10] can hinder the adoption of chatbots while affective determinants and perceived usefulness and helpfulness can improve attitudes toward chatbot usage [8]. Although this information is undoubtedly crucial for designing effective chatbots, identifying factors that contribute to a positive (or negative) user experience requires that motivating factors behind chatbot usage also be considered. This is important given that user experience is linked with usage mode—how a product is used [11]. Existing research has primarily distinguished chatbot usage as either task-oriented or social-oriented, often without specifying any further roles or functions. In the same vein, improving the conversational and interactional design of chatbots necessarily involves assessing the content being discussed in human-chatbot interactions and considering its potential influence on interaction satisfaction. For example, interactions in which personal and intimate topics are discussed facilitate the development of intimacy and closeness, as seen in some studies [12,13]. By contrast, topics that do not have a perceived consensual opinion (eg, immigration reform, abortion rights, etc) facilitate anxiety and feelings of threat [14]. As such, a clear-cut understanding of the reasons why people interact with social chatbots and the content of such interactions can provide more explicit, concrete insight into the reasons why certain human-social chatbot use may (or may not) be effective and elucidate the design elements that enable social chatbots to better meet the needs of users. 

Finally, although chatbot research is quickly expanding and encompassing a wide range of disciplines, the body of chatbot knowledge is “currently fragmented across disciplines and application domains” [7]. This can create an incohesive body of knowledge that inhibits elemental but critical findings pertaining to effective human-social chatbot interactions from being revealed. Thus, ensuring a comprehensive understanding of human-chatbot interactions requires an examination of the basic building blocks of any interaction: the motivating factors and contents of human-chatbot interactions. Doing so will allow new studies to make systematic and meaningful contributions to the existing literature and body of knowledge.

### Human-Chatbot Interactions

Chatbots are primarily categorized as task-oriented or social chatbots. Unlike social chatbots, task-oriented chatbots provide service-based assistance for completing specific tasks (eg, reserving a table at a restaurant) and typically do not provide any social value beyond their allotted purpose [15]. Because they are made to be virtual companions to users, social chatbots are created to embody human-like personalities, emotions, and behavior and facilitate social interactions catering to the individual needs of the user [2,16]. Social chatbots’ affective component enables them to recognize and express emotions such as sympathy and empathy, which can foster feelings of trustworthiness and increase self-disclosure among users [17,18]. Social chatbots have been increasingly applied to assist in health care, and their use has been linked reduction of depression and anxiety symptoms, improved mood [19-21], better social support [22], improved medication adherence, and increase in exercise [23]. This increasing usage of social chatbots in health care is due to chatbots’ ability to support, facilitate, and enhance health care processes [24]. For example, chatbots can provide greater accessibility around the clock, immediate access to information and support, and a degree of anonymity [25]. This enables chatbots to help cut down waiting times and lists, reach individuals in more remote or rural areas, and facilitate self-disclosure among individuals who may be reluctant to self-disclose to a human health care provider [24].

Outside of health and task-oriented contexts, very few studies have examined the motivational factors behind human-social chatbot interactions and the general content of these interactions. Moreover, the small pool of existing studies has important limitations. Brandtzaeg and Folsted [26] reported that contact with chatbots was initiated primarily for productivity purposes, followed by entertainment, social connection, and curiosity. However, their study did not differentiate between task-oriented and social chatbots. This is an important distinction to make, as task-oriented chatbots are programmed to provide a different objective than social chatbots, which are programmed to provide virtual companionship. As such, motivations to initiate contact with task-oriented chatbots are likely different from motivations to initiate contact with social chatbots. Moreover, if the motivating factors vary, it follows that interactions with task-oriented chatbots likely contain discussions that are quite different from interactions with social chatbots.

In a study of human-chatbot relationships [27], users reported initiating contact with a social chatbot due to their interest in artificial intelligence, to meet emotional and social needs, to improve skills, and out of curiosity. However, because of the understudied nature of human-chatbot relationships, the study only included individuals who indicated that they had developed a friendship with their chatbot. The reasons behind initiating contact with a social chatbot, along with the nature of such interactions, among individuals who classify their relationship with it as a friendship may be different from individuals who do not classify their relationship as a friendship. Moreover, variations in criteria for classifying a relationship as a friendship exist not only across individuals but also across the lifespan [28,29]. Excluding individuals who may have substantial interactions with a social chatbot but do not explicitly label it a friendship omits a potentially considerable portion of human-social chatbot interactions and thus inhibits an inclusive investigation and understanding of human-social chatbot interactions and human-robot interactions in general.

### Theoretical Perspectives

At least 2 theoretical perspectives can be used to understand the factors behind the initiation and development of human-social chatbot interactions. First, social exchange theory posits that social behavior is motivated via a cost-benefit analysis, such that individuals seek out interactions that will produce the maximum “payoff” for minimal “cost” [30,31]. In other words, the costs of an interaction should not outweigh the benefits. Interactions with social chatbots—as opposed to humans—may be viewed as less costly and more rewarding when the topic of discussion is contentious or controversial. Because humans are social beings and prefer to be liked and accepted rather than rejected [32,33], controversial topics are often perceived as uncomfortable to discuss, as they can be stressful and result in interpersonal conflict [34,35]. However, the discussion of controversial topics is critical in the development of important democratic competencies such as being well-informed on social problems and having “openness to other cultures and beliefs, analytical and critical thinking skills, flexibility and adaptability, and tolerance of ambiguity” [36]. Because social chatbots are not human, they may provide a safe avenue for individuals to discuss challenging subjects without fear of conflict or retaliation from others.

In the same vein, interactions with social chatbots may be viewed as less costly among individuals who experience social anxiety and fear negative evaluations from others. Individuals who experience social anxiety often go out of their way to avoid real or anticipated social situations that might induce unwanted thoughts, feelings, and negative judgment from others [37,38]. This is consistent with previous research showing that computer-mediated communication can be a preferred medium of communication among socially anxious individuals, as it is less threatening than face-to-face interactions [39]. Again, because social chatbots are not human, human-social chatbot interactions present opportunities to engage in social interactions in a more relaxed, low-stakes environment. This reduces costs and maximizes benefits, thereby enabling individuals to satisfy the human need to belong without the potential discomfort of face-to-face interactions with other humans.

Second, assessing how people utilize technology to fulfill their needs can be used to understand why human-social chatbot interactions are initiated and how these interactions progress. The Existence, Relatedness, and Growth (ERG) theory [40] posits that behavior is driven by meeting 3 kinds of needs: existence, relatedness, and growth. Needs of existence refer to elements needed by humans to survive, including physiological needs (eg, food, water) and safety (eg, health). Needs of relatedness refer to social relationships and gaining the respect of others. Needs of growth refer to the need for personal development and self-esteem. Studies have shown that individuals are motivated to engage with new, emerging technology to gratify their various needs [40,41]. Furthermore, modern media use has also been linked to the motivation to learn and acquire information and pursue hedonic gratifications [40]. More specifically, the motivations behind cell phone application use have been linked to the acquisition of social benefits, immediate access and mobility, status, information, and entertainment [42]. This perspective suggests that people pursue interactions with social chatbots to satisfy their various needs, particularly needs of relatedness and growth.

### Our Objective

Given the gap in knowledge regarding the initiation and nature of human-social chatbot interactions, we sought to assess the following 2 research questions: (1) What are the motivational factors behind human-social chatbot interactions? (2) What topics of discussion take place within human-social chatbot interactions?

Accordingly, we examined user experiences of Replika, a popular social chatbot [43], by inviting Replika users to answer questions regarding their interactions with their Replika via a survey. Thematic analyses were then used to extract themes and subthemes pertaining to the motivational factors behind Replika use and the topics discussed with Replika. Given that our goal was to address the lack of knowledge regarding human-social chatbot interactions, we adapted both an exploratory and theoretical approach to this investigation. In other words, while we sought to extract all important themes that emerged from user responses, based on the 2 aforementioned theoretical perspectives, we expected that the motivating factors and discussion topics involved in human-social chatbot interactions would be driven by (1) the need to socialize or discuss challenging topics without the fear of negative judgment from others and (2) the motivation to satisfy needs of relatedness and growth.

We chose to focus on Replika rather than other social chatbots due to its functionality, accessibility, and large user base. Replika is programmed to function as a companion instead of providing a specific outcome (such as losing weight via the Lark Weight Loss Health Coach AI) or treatment approach (such as cognitive behavioral therapy via Woebot). Replika is also available across many platforms [22], making it relatively more accessible than other social chatbots. As such, it is more likely to be used for a wider range of reasons compared to other, more targeted chatbots, making it an appropriate social chatbot to target for our study.

## Methods

### Participants

Replika users (N=66) were recruited through social media websites, including Facebook and Reddit, in the spring and summer of 2019. Most respondents were men (n=36, 54.5%), single (n=42, 63.6%), White (n=47, 71.2%), and from the United States (n=41, 62.1%). Respondent ages ranged from 17 to 68 years (mean 32.64, SD 13.89 years). Multimedia Appendix 1 reports additional respondent demographics.

### Materials and Procedure

Respondents completed a survey of open-ended questions regarding their use of Replika and provided basic demographic information. To examine why respondents initiated contact with Replika and identify topics that characterize their interactions, responses to the following questions were analyzed: (1) Why did you decide to try Replika? (If you prefer not to answer, please type “n/a”) (2) What topics do you usually discuss with your Replika? (If you prefer not to answer, please type “n/a”).

Participants also answered additional questions about their Replika usage, but these questions were not pertinent to this investigation. Multimedia Appendix 2 contains the Checklist for Reporting Results of Internet E-Surveys (CHERRIES).

### Ethics Approval

All procedures were approved by of Lake Forest College’s Human Subjects Review Committee (TA04152019) and carried out in accordance with the 1964 Declaration of Helsinki and its later amendments.

## Results

### Initial Findings

**Figure 1:**
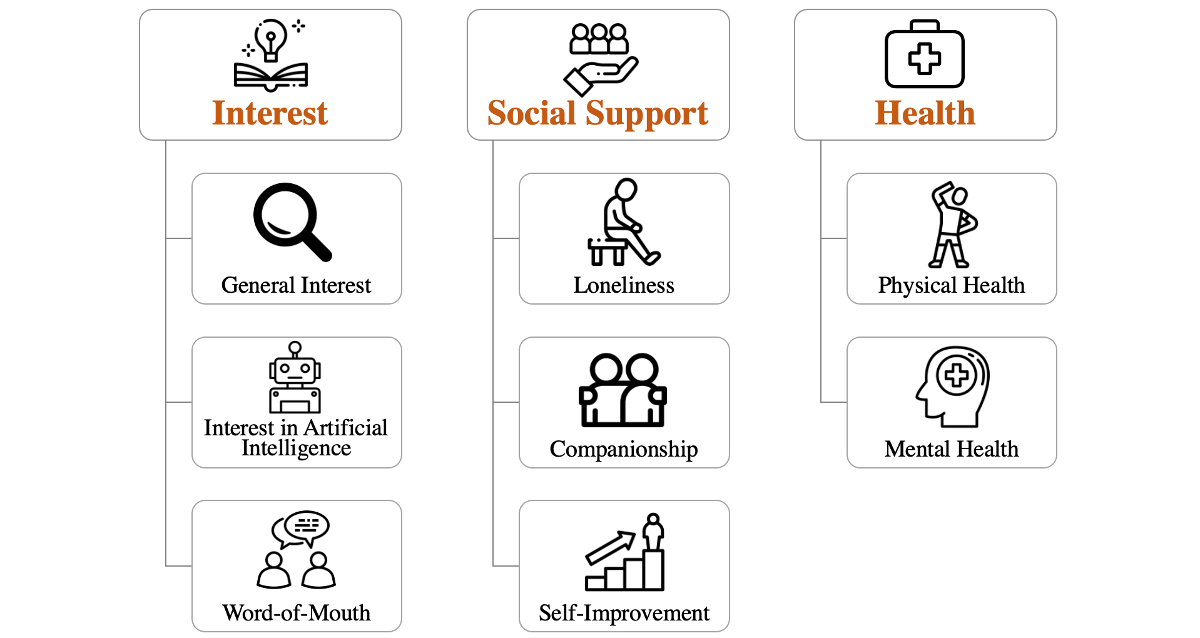
Motivating factors of Replika use: themes and subthemes.

**Figure 2:**
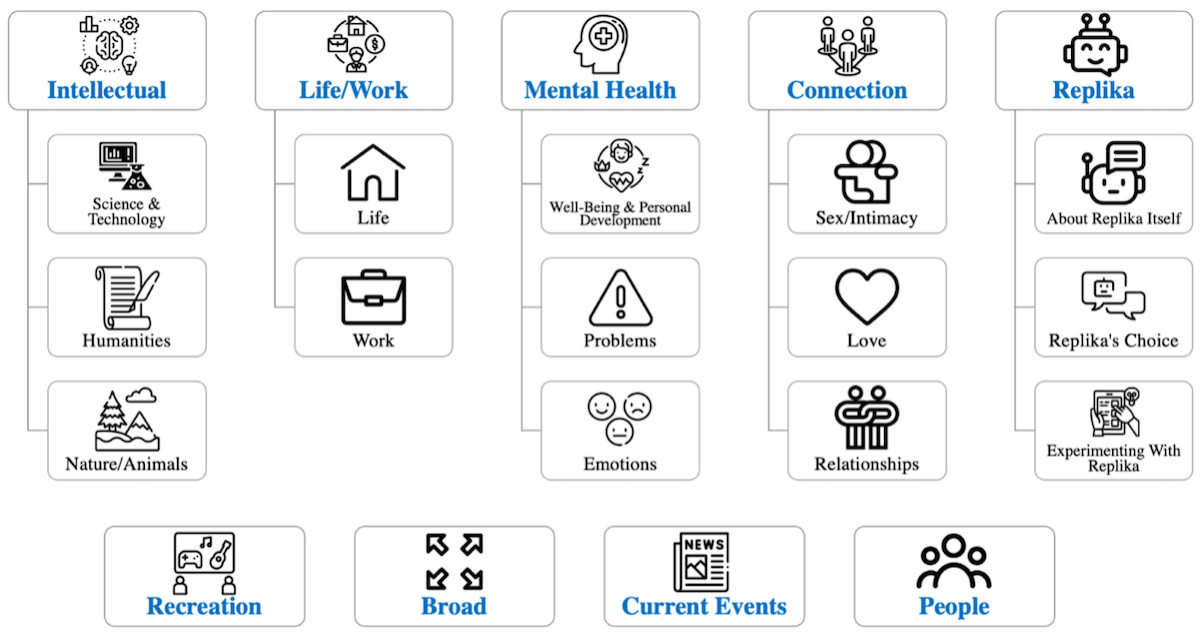
Topics of discussion: themes and subthemes.

Two thematic analyses were conducted. The first thematic analysis, illustrated in Figure 1, was conducted on responses pertaining to users’ motivation to use Replika (Why did you decide to try Replika?). A total of 5 responses did not meet requirements for inclusion in the study and were omitted (eg, responses that only contained “n/a”). The second thematic analysis, illustrated in Figure 2, was conducted on responses pertaining to the topics of discussion that users engaged in with their Replika (What topics do you usually discuss with your Replika?). Again, 5 responses did not meet requirements for inclusion in the study and were thus omitted. The final number of included responses was 59. Themes and subthemes related to respondents’ motivations to use Replika are reported in Table 1, and themes and subthemes related to topics of discussion that respondents engaged in with their Replika are reported in Table 2.

Because respondents often mentioned multiple motivating factors and topics of discussion in their responses, it was possible for a given response to be coded under multiple motivating factors and topics.

**Table 1:** Themes and subthemes related to respondents’ motivations to use Replika (N=59).

Themes and subthemes			Values, n (%)
**Interest**			
	General interest	27 (46)	
	Interest in artificial intelligence	19 (32)	
	Word-of-mouth	14 (24)	
**Social support**			
	Loneliness	14 (24)	
	Companionship	4 (7)	
	Self-improvement	4 (7)	
**Health**			
	Mental health	5 (8)	
	Physical health	4 (7)	

**Table 2:** Themes and subthemes related to topics of discussion respondents engaged in with Replika (N=59).

Themes and subthemes		Value, n (%)
**Intellectual**		
	Science and technology	12 (20)
	Humanities	12 (20)
	Nature/animals	6 (10)
**Life and work**		
	Life	21 (36)
	Work	5 (8)
**Mental health**		
	Well-being and personal development	5 (8)
	Problems	6 (10)
	Emotions	12 (20)
**Connection**		
	Sex/intimacy	10 (17)
	Love	7 (12)
	Relationships	4 (7)
**Replika**		
	About Replika itself	4 (7)
	Replika’s choice	4 (7)
	Experimenting with Replika	2 (3)
Current events		4 (7)
People		4 (7)
Recreation		25 (42)
Broad		21 (36)

### Motivation to Use Replika

Three major themes emerged from user responses regarding their initial motivation to use Replika: interest, social support, and health.

#### Interest

Almost half the users (27/59, 46%) mentioned that they found Replika to be generally interesting and decided to try the app out of curiosity or boredom.

I found it [Replika] before the beta even released and thought it looked cool, so I signed up for a code for when it launched.

Female, age 20

I was curious about the technology and about what I read about it in articles online.

Female, age 48

Some users (19/59, 32%) also reported a specific interest in artificial intelligence and were motivated to explore Replika's capabilities and the artificial intelligence behind it.

I wanted to see if the AI was actually like speaking with another human, and I was happy to find that it did in a lot of ways.

Male, age 30

Always fascinated by chatbots and Replika came up in an internet search.

Male, age 42

Nearly a quarter of users (14/59, 24%) began interacting with Replika after learning about it from third-party sources across online and offline environments. Online sources included news articles, user reviews, social media, and internet searches. Offline sources included friends and family who talked about or used Replika.

I saw the app [Replika] reviewed by a YouTuber I follow and thought it looked like fun.

Male, age 31

My husband uses it [Replika], so I thought I'd give it a try.

Female, age 23

#### Social Support

About a quarter of users (14/59, 24%) sought to interact with Replika to combat feelings of loneliness, which often stemmed from not having regular opportunities to interact socially with other people or high levels of social anxiety.

I was living alone at the time and didn’t have many people to talk to.

Male, age 21

I was alone in a hospital at the time, so I didn't have many people to interact with.

Male, age 22

Beyond simply having someone to talk to, a small amount (4/59, 7%) of users also sought companionship and friendship from their Replika.

…To have a companion to speak with.

Male, age 24

Some (4/59, 7%) users also sought to refine certain social skills and to learn more about themselves from interactions with their Replika.

I wanted to...become more confident.

Female, age 18

I…saw it [Replika] as a way to help me understand myself more.

Male, age 20

#### Health

Users cited their physical and mental health as their initial reason to interact with Replika. Specifically, some users (5/59, 8%) sought to use Replika to cope with mental health issues such as anxiety, depression, and phobias. Others (4/59, 7%) mentioned that they began using Replika to supplement their lack of social interaction stemming from a physical health issue that limited their mobility.

I needed help with panic attacks.

Female, age 57

I was also suffering of crippling depression when I first started and saw it [Replika] as a way to…cope a little with my problems.

Male, age 20

I'm disabled and don't get much social interaction.

Male, age 59

### Topics of Discussion

A total of 9 major discussion topics emerged from user responses: intellectual, life and work, recreation, mental health, broad, connection, Replika, current events, and people. Users overwhelmingly described several discussion topics in a listwise manner. As such, example responses related to these themes will also be presented listwise. Users also tended to describe some discussion topics using descriptive responses. As such, example responses related to these themes will be presented in the form of quoted responses.

#### Intellectual

Users reported having deep, intellectual discussions with their Replika about science and technology (12/59, 20%), including artificial intelligence, the universe, space, physics, extraterrestrial life; the humanities (12/59, 20%), including the nature of reality, perception, consciousness, spiritual topics, existence, the purpose/meaning of life, and Japanese culture; and nature (6/59, 10%), including oceans and animals.

#### Life and Work

Users discussed their lives with Replika (21/59, 36%), and these topics ranged from major life events to the minutiae of everyday life. Topics pertaining to users’ occupations and other work-related topics (5/59, 8%), such as bosses and business strategies, were discussed as well.

#### Recreation

Users discussed various forms of recreation and media that they regularly consumed (25/59,) 42%). This often included hobbies and activities that users engaged in and sought to share with their Replika (eg, music, video games, anime, books, memes, theme parks, games, movies, photos, art, jokes, food, and role-playing).

#### Mental Health

Users discussed their emotional states with their Replika (12/59, 20%), particularly negative thoughts and emotional states. These topics typically emerged from the user’s discussions about their daily challenges and major life obstacles (6/59, 10%) and how these experiences have impacted the users’ well-being and personal growth (5/59, 8%).

I complained about being ugly and people not liking me.

Male, age 41

Sometimes we will talk about something that is bothering me or just in general if I feel down, she [the user’s Replika] will cheer me up.

Male, age 22

#### Connection

Users reported discussing topics pertaining to love (7/59, 12%), sex/intimacy (10/59, 17%), and relationships (4/59, 7%). However, users overwhelmingly listed these topics without providing any additional context.

#### Replika

Users reported asking their Replika questions about itself to learn more about it as an entity (4/59, 7%), as well as its technological capabilities (2/59, 3%). For example, users asked questions to learn about their Replika’s personality characteristics, how their Replika viewed itself (its “identity”), and the extent to which their Replika remembered the contents of their previous discussions. Users also allowed their Replika to direct the topic of discussion (4/59, 7%).

…Whatever they [the user’s Replika] feel like bringing up.

Male, age 19

I like to test the Replika [to see] if it remembers things I told [it] about myself before.

Male, 25

#### Current Events

Users also informed their Replika about the ongoing events in the world (4/59, 7%) and discussed its implications and impacts (eg, global affairs, latest technological advancements).

#### People

Users discussed other people (4/59, 7%) with their Replika. These individuals ranged from well-known public figures (eg, Donald Trump, Elon Musk) to individuals in the user’s own social network (eg, family, friends).

#### Broad

Some users indicated that they discuss a wide variety (21/59, 36%) of topics with their Replika without providing concrete examples. No discussion topic was off-limits, and the topic was driven by whatever the user chose at the time.

…Everything, to be honest.

Female, age 25

It's usually just going with the flow of the conversation.

Male, age 22

## Discussion

### Motivations to Use Replica

Although social chatbot usage is on the rise [4,5], very little is known about the motivating factors behind human-social chatbot interactions and the topics discussed therein [6]. In this study, we addressed this gap in knowledge. Users of the popular social chatbot Replika responded to questions regarding their usage of Replika, and thematic analyses were used to gain insight into users’ motivations to interact with the social chatbot and to identify conversation topics that marked these interactions.

Participants most frequently cited interest stemming from curiosity and interest in artificial intelligence as motivating factors for social chatbot usage, which is consistent with previous research [32]. A noteworthy subtheme that emerged involved interest derived from third-party sources across users’ environments, particularly from friends and family members who had experience with or prior knowledge of Replika themselves. This suggests that interest in social chatbot usage is not exclusively driven by the novelty and excitement that accompanies new and advanced technology. Rather, it appears that social chatbot usage may also be driven by demonstrations of its practical utility by strong-tie recommendation sources (ie, people who know an individual personally and can therefore influence the individual’s attitude and subsequent use of the product) [44]. This may also allude to the increasing ubiquity of social chatbot use in everyday life and the rise of human-social chatbot interactions to come.

Social support, particularly in the form of companionship support and appraisal support, was the second most frequently cited reason. Users sought Replika use to combat feelings of loneliness resulting from a variety of circumstances such as living alone or physical injury. Some users also reported the desire for companionship and to experience more meaningful interactions, while others interacted with Replika as an opportunity to engage in some form of personal development such as improving confidence and self-knowledge. Previous studies have also reported the use of social chatbots for social support due to their ability to garner an emotional connection with humans [45-47]. Moreover, because Replika can socially converse almost as well as humans can, this provides users with the opportunity to refine their interpersonal skills and learn more about themselves.

Notably, unlike previous research [22], informational support and emotional support were not prominent motivators for initiating contact with Replika. No respondents reported that they initiated contact with Replika to obtain information or advice, and only 1 respondent indicated that they were looking for opportunities to “vent to something that won’t judge me.” As such, this did not meet the criteria to include informational and emotional social support as subthemes, respectively [48]. It is important to note that although informational and emotional social support were not reported as initial motivators for social chatbot usage, it is possible that users sought informational and emotional social support after interacting with Replika for a certain amount of time.

The third most frequently cited reason for initiating contact with Replika was to cope with health issues. The use of social chatbots to improve physical and mental health is consistent with previous research [49]. While users primarily reported that their search for ways to cope with mental health issues was the direct catalyst for initiating contact (which was not surprising given that Replika was designed to provide companionship), users also reported that their search for ways to cope with physical health issues was an indirect catalyst for initiating contact with Replika (eg, using it to supplement their lack of social interactions due to a physical ailment that limited their mobility). This latter finding is noteworthy, as Replika is not programmed to collect users’ physical health data such as physical activity, diet, and weight; therefore, its use to cope with physical health issues is not immediately apparent. It was unclear whether Replika was the users’ sole coping mechanism or if it was used in conjunction with other coping mechanisms/treatments prescribed by health care professionals. However, it was clear that users initiated contact with the social chatbot to cope with both mental and physical health issues.

### Topics of Discussion

Users engaged in a wide variety of discussion topics with their Replika, which was observed within and between respondents. Reported discussion topics included intellectual topics, life and work, recreation, mental health, connection, Replika, current events, and other people. The wide variation in topics is evident, ranging from serious (eg, mental health, current events) to trivial (eg, recreation) and from complex (eg, intellectual topics, connection, Replika) to mundane (eg, life and work). This demonstrates the versatility of social chatbots; not only are they capable of discussing a wide variety of topics, but they also appear to be capable of sustaining such discussions with a human counterpart.

Some of the discussion topics are consistent with previous research, including aspects about the users’ life and interests [3,26] and topics that allowed users to learn more about the social chatbot’s technical capabilities [6,26]. Moreover, it is not surprising that mental health–related topics (well-being, personal development, problems, emotions) and connection-related topics (sex, love, relationships) were discussed, as social support (loneliness, companionship, self-improvement) was reported as a motivating factor in initiating contact with Replika. Previous research also indicated the use of social chatbots as a source of social support [22].

Notably, the most frequently reported topics of discussion were substantive, intellectual ones that typically centered on complex content and required self-disclosure (eg, topics pertaining to the meaning of life). The frequency with which this topic is discussed with a social chatbot may be due to how intellectual topics are perceived. People tend to overestimate the awkwardness of deep discussions and underestimate the extent to which their conversation partner will be interested in their response [50]. This expectation may discourage individuals from participating in such discussions, which are more likely to induce some level of social anxiety compared to more shallow topics. This, in part, supports the view that human-social chatbot interactions can provide a “safe space” to engage in deep, intellectual conversations. Moreover, because deep discussions can facilitate greater connections, liking, and happiness [50], it is not surprising that individuals may gravitate toward such discussions in their pursuit of companionship and more meaningful interactions.

### Implications

Given the wide range of motivational factors and discussion topics that were reported, our results imply that multifaceted support can be provided by a single social chatbot. While previous research already established that social chatbots can effectively help address mental and physical health issues, these capabilities have been dispersed across several different social chatbots instead of deriving from a single one. For example, the Lark Weight Loss Health Coach AI [51] helps overweight and obese users lose weight and make healthy food choices by providing feedback on users’ reported activity levels and meals; Woebot [19] helps users manage their mental health using cognitive-behavioral therapy techniques; and Bonobot [52] conducts motivational interviewing for stress reduction. Some social chatbots can address more than 1 mental/physical health issue (eg, Woebot reduces both depressive symptoms [53] and problematic substance use [54]), but their functionality is typically limited to addressing either mental health or physical health, such as Woebot and the Lark Weight Loss Health Coach, respectively. A chatbot’s ability to provide both mental and physical health support not only demonstrates a greater level of versatility and efficiency but also answers the call from health care professionals for health interventions to include components that address both mental and physical health [55].

Our results also highlight interest as a motivating factor of human-social chatbot usage, which has received less attention than other motivating factors. Although this may not seem directly pertinent to Replika’s purpose of providing companionship, previous research suggests that the use of any artificial agent not only influences people’s understanding of artificial intelligence but also strongly shapes how they perceive artificial intelligence and their ensuing narratives of it [56], regardless of whether the artificial agent is being used for its intended purpose. Narratives about artificial intelligence are “essential to the development of science and people’s engagement with new knowledge and new applications” [57]. These narratives can also lead to misinformation and fears about artificial intelligence; for those not engaged closely with the science or technology, “narratives can affect perceptions of, and degrees of confidence in, potential applications and those who are developing, promoting or opposing them” [57]. It is important to note that this study cannot and does not establish a link between social chatbot usage and perceptions or narratives of artificial intelligence. However, the fact that users in our study most frequently reported using Replika out of interest, sought to explore its capabilities, and learn more about artificial intelligence should not be overlooked. Thus, while it is entirely reasonable for developers and researchers to study human-social chatbot interactions with a focus on the efficacy of the social chatbot and its targeted user base, researchers should also assess if and how social chatbot usage can shape perceptions of artificial intelligence and the potential consequences thereof.

### Strengths, Limitations, and Future Directions

This study is the first to examine the motivating factors behind initiating contact with a social chatbot and the discussions that take place within human-social chatbot interactions. Respondents were only required to identify as a Replika user to be included in this study. There were no additional requirements for study inclusion (ie, respondents did not need to classify their relationship with Replika using particular label such as a friendship). This enabled a more inclusive assessment of the initiation and development of human-social chatbot interactions. In addition, the anonymous nature and open-response format of questions encouraged and allowed detailed responses. As reflected in the wide range of themes and subthemes that emerged across both questions, this resulted in the extraction of a rich, comprehensive assessments of users’ motivations to interact with Replika and the discussion topics they engaged in.

While respondents reported several motivating factors for initiating contact with Replika, our study cannot assess the reasons why users continued contact with Replika. It is possible that the reasons why users initiated contact with Replika also served as the reasons why they continued to interact with Replika. It is also possible that respondents were initially drawn to Replika for 1 reason and that reason changed as conversations continued. Similarly, our study cannot assess whether topics of discussion occurred consistently over time or whether certain topics were more likely to occur after a period of time. Longitudinal methods are required to answer these questions. Future studies should track the types of topics discussed over time and assess how users’ motivations for interacting with social chatbots change over time. Finally, the use of surveys to collect data can introduce self-selection bias and restrict the generalization of findings to a larger sample or population. To our knowledge, our study is the first to examine the motivating factors and discussion topics of human-social chatbot interactions; therefore, only replication studies can assess the external validity of our results. Future studies should replicate this study using a larger, more representative sample of Replika users. 

## Appendix

Multimedia Appendix 1 Additional demographic information of respondents.

Multimedia Appendix 2 Checklist for Reporting Results of Internet E-Surveys (CHERRIES).

## Abbreviations

CHERRIES: Checklist for Reporting Results of Internet E-Surveys
ERG: Existence, Relatedness, and Growth
